# Emergency Department Blood Pressure Management in Type B Aortic Dissection: An Analysis with Machine Learning

**DOI:** 10.5811/westjem.25005

**Published:** 2025-05-05

**Authors:** Nelson Chen, Jessica V. Downing, Jacob Epstein, Samira Mudd, Angie Chan, Sneha Kuppireddy, Roya Tehrani, Isha Vashee, Emily Hart, Emily Esposito, Rose Chasm, Quincy K. Tran

**Affiliations:** * University of Maryland School of Medicine, Baltimore, Maryland; † Research Associate Program in Emergency Medicine and Critical Care, University of Maryland School of Medicine, Department of Emergency Medicine, Baltimore, Maryland; ‡ University of Maryland School of Medicine, Department of Emergency Medicine, Baltimore, Maryland; § University of Maryland School of Medicine, Program in Trauma, The R. Adam Cowley Shock Trauma Center, Baltimore, Maryland; || University of Maryland Medical Center, Critical Care Resuscitation Unit, Baltimore, Maryland

## Abstract

**Background:**

Acute aortic dissections (AAD) have a high morbidity and mortality rate. Treatment for type B aortic dissection includes strict systolic blood pressure (SBP) and heart rate (HR) control per the American Heart Association (AHA) guidelines. However, predictors of successful emergency department (ED) management of SBP have not been well studied.

**Methods:**

We retrospectively analyzed the records of adult patients presenting to any regional ED with type B AAD between 2017–2020 with initial SBP >120 mmHg and HR >60 beats per minute (bpm) and were subsequently transferred to our quaternary center. Primary outcome was SBP <120 mmHg based on both the 2010 and 2022 AHA guidelines and HR <60 bpm (based on the 2010 guideline), or HR <80 (2022 guideline). We used random forest (RF) algorithms, a machine-learning tool that uses clusters of decision trees to predict a categorical outcome, to identify predictors of achieving HR and SBP goals prior to ED departure, defined as the time point at which patients left the referring ED to come to our institution.

**Results:**

The analysis included 134 patients. At the time of ED departure, 26 (19%) had SBP <120 mmHg, 96 (67%) received anti-impulse therapy, and 40 (28%) received beta-blocker or vasodilator infusions specifically. The RF algorithm identified higher triage SBP and treatment with intravenous labetalol as the top predictors for SBP >120 mmHg at ED departure, contrary to AHA guidelines. Pain management with higher total morphine equivalent unit, as well as shorter time to computed tomography as predictors for HR <60 bpm and <80 bpm, were in concert with AHA guidelines.

**Conclusion:**

Many patients with type B AAD did not achieve hemodynamic parameters in line with 2010 or 2022 AHA guidelines while being in the ED prior to transferring to a quaternary care center for further evaluation and management. Patients with higher heart rate and systolic blood pressure on ED arrival were less likely to achieve goals at the time of departure from the referring EDs. Those receiving more pain medications prior to transfer were more likely to meet certain AHA goals.

## INTRODUCTION

Acute aortic dissection (AAD) occurs when there is a tear in the intima of the aorta resulting in the formation of a false lumen that might cause organ ischemia. An aortic dissection is classified as type A when the dissection flap originates in the ascending aorta proximal to the subclavian artery; type B occurs when the dissection originates in the descending aorta, after the take-off of the subclavian.^1^ Up to 35% of people who experience one of these acute aortic syndromes die immediately.^2^,^3^ Overall, the one-year mortality rate is 90%,^3^ and approximately 25% of patients die during hospitalization. While type B aortic dissections are relatively uncommon, patients may have severe complications when left untreated, the most serious being rupture of the aorta.^4^

Type A dissection is considered a surgical emergency, while the initial management of a type B dissection is traditionally medical (although when end-organ damage occurs, a type B dissection becomes a surgical emergency).^5^ The American Heart Association (AHA) has published and routinely updates guidelines to provide a framework for the management of acute aortic dissections. The most recent iteration of these guidelines was updated in 2022 and suggests the use of medications to acutely lower and maintain patients’ systolic blood pressure (SBP) <120 mmHg “or to the lowest BP that allows end-organ perfusion,” and heart rate (HR) between 60–80 beats per minute (bpm).^2^ (These goals were modifications from the prior guideline’s stricter recommendations of HR <60 bpm.^6^) For patients without contraindications, intravenous (IV) beta-blockers (often esmolol, metoprolol, or labetalol) are recommended as first-line therapy, with IV vasodilators (such as nicardipine, clevidipine, or nitroprusside) added on if BP control remains suboptimal after beta-blockers have been started and appropriately titrated. It is recommended that patients with contraindications to beta-blockers be treated first line with non-dihydropyridine calcium channel blockers (CCB) for HR control. Pain management is also recommended, as uncontrolled pain may worsen tachycardia and hypertension. The goal of this “anti-impulse” therapy is to reduce the shearing force on the wall of the aorta while maintaining adequate end-organ perfusion.^7^–^9^

Emergency physicians (EP) often are the first to triage and diagnose patients who present with AADs and initiate medical management before admitting or transferring them to tertiary care hospitals for continued treatment. With the high morbidity and mortality of this disease, early and effective treatment is necessary to prevent complications.^10^ However, data suggests EPs may be falling short of this goal. A prior study showed that among all patients who had “suspected acute aortic dissection” and were transferred to a tertiary care center via air transport, most of these patients’ initial vital signs upon leaving the referring EDs were outside the target range recommended by the 2010 AHA at the time.^11^ This study showed that only 25% of patients attained a SBP <120 mm Hg and 64% a HR <80 bpm by the time of their departure from the ED.^11^ Many of the patients in this group did not receive any antihypertensive (AHT) therapies prior to transfer to a higher level of care. Similarly, another study showed that among 168 patients with acute aortic disease who underwent ED-to-ED transfer to a quaternary care center, 19% and 9% of patients would achieve SBP <120 mmHg or HR <60 bpm, respectively, when they left the referring EDs.^12^

In this study we aimed to investigate the rate at which patients transferred directly from an ED to a quaternary hospital for the management of type B dissection achieve SBP and HR measurements within the parameters recommended by the AHA during patients’ ED stay prior to transfer. We also aimed to identify patient characteristics and treatment factors associated with meeting those targets. Having more information regarding any effective modality for treating patients with type B aortic dissection would provide emergency clinicians with further information to improve patient care.

**Population Health Research Capsule**

What do we already know about this issue?

*Type B aortic dissections are hypertensive emergencies managed via rapid control of heart rate (HR) and systolic blood pressure (SBP).*

What was the research question?

*What percentage of ED patients with type B dissection achieve recommended HR and SBP prior to transfer? What factors predict success?*

What was the major finding of the study?

*11% of patients met 2022 guidelines for HR and SBP control. Older age and higher triage HR and SBP were predictive of poor BP control in the ED.*

How does this improve population health?

*By highlighting the low rate of HR and SBP control in EDs, our findings emphasize the importance of early and aggressive medical management to improve outcomes.*

## METHODS

### Study Setting

This was a retrospective study of adult patients transferred from any ED to the critical care resuscitation unit (CCRU) at the University of Maryland Medical Center (UMMC) from January 1, 2017–December 31, 2020. The policy at our institution mandates that all patients from other hospitals who require urgent surgical evaluation, including patients with any AAD, be transferred to the CCRU first, before going to any other inpatient units of the hospital. Thus, all patients being transferred to UMMC for management of acute aortic diseases are first admitted to the CCRU for initial stabilization and management. Collaborating with the cardiac surgeons or vascular surgeons at our institution, the CCRU clinicians perform acute medical management and resuscitation for patients with a variety of medical and surgical emergencies across specialties. Details regarding the clinicians, nursing staffing, and model of the CCRU have been published elsewhere.^14^ The CCRU was designed to have the capabilities of a variety of subspecialty intensive care units (ICU), including cardiac surgery and cardiovascular ICUs.

The UMMC is home to a multidisciplinary Center for Aortic Disease composed of specialists in cardiac and vascular surgery, vascular medicine, and cardiology. Cardiac and vascular surgery specialists are available 24/7 for the initial evaluation and management of patients transferred for acute aortic syndromes. The treatment plans of patients with type B aortic dissections are primarily directed by the vascular surgery team, which evaluates patients immediately upon their arrival at the CCRU. Patients with evidence of end-organ malperfusion or enlarging dissections may be considered candidates for operative or endovascular repair, including physician-modified endovascular grafting.^17^

Following initial stabilization and the identification of an available appropriate bed, patients are transferred to another ICU, often the surgical ICU or cardiac surgery ICU or another appropriate inpatient unit at UMMC. During times characterized by extremely limited bed availability, patients may remain in the CCRU throughout their initial treatment period and be successfully transferred to an intermediate or “step-down” unit following transition from continuous infusions for anti-impulse therapy and onto oral AHTs.

### Patient Selection

All patients who were transferred from an ED to the CCRU between 2017–2020 for type B ADDs were eligible for inclusion in this study. We excluded patients with missing records from the referring ED such as vital signs at ED triage and at the time of departure from the referring ED. Patients with SBP <90 mmHg and a heart rate <60 bpm at the time of ED triage were also excluded. The study was approved by the institutional review board at our institution (HP-00084554).

### Data Collection and Management

We collected patient data by reviewing our health system’s electronic health record system (EHR) (Epic Systems Corporation, Verona, WI). If the referring EDs did not use Epic or the records were not available immediately, the investigator extracted data from the paper version of the documents that accompanied patients when they were transferred to our institution and subsequently scanned into their EHR. Data was extracted into a standardized Excel spreadsheet (Microsoft Corporation, Redmond, WA). Before data extraction, all investigators, who were blinded to the hypothesis, were trained by the primary investigator for data extraction, using sets of 10 patient charts. Training data was compared between investigators and senior investigators until agreement reached 90%. During data collection, up to 5% of data was randomly checked by a senior investigator for accuracy. Our protocol adhered to 7 of 8 of the recommendations outlined by Worster and Bledsoe (except case selection criteria).^13^

We collected information regarding patient demographics, past medical history, social history, and home medications. In addition, we recorded clinical ED data including all SBP and HR measurements recorded throughout their entire ED stay. We extracted data regarding the type, dose, administration route, and administration time (as documented by an ED registered nurse [RN] in the medical record) of beta-blockers, CCBs, vasodilatory medications, and pain medications. If a medication was ordered by the clinician but not documented as given by the RN, that medication was not extracted. We also collected the timing and results of all computed tomography and laboratory values such as serum lactate and creatinine levels, as well as ED triage and departure times.

### Outcome Measures

Our primary outcome was SBP <120 mm Hg at the time of departing the referring EDs, as is consistent with the recommendation by both the 2010 and 2022 AHA guidelines. Secondary outcomes included HR <60 bpm (2011 AHA guideline) and HR <80 bpm (2022 AHA guideline).

### Data Analysis

We did not perform a sample size calculation for this study due to its exploratory nature and because the outcomes were the prevalence of patients achieving guidelines’ recommendation. Histograms of continuous variables were examined for their pattern of distributions and expressed as mean (+/− standard deviation) or median (interquartile range [IQR]) as indicated and analyzed using the Student *t*-test or Mann-Whitney U test as indicated. Categorical variables were expressed as N, and percentages and were analyzed with the chi-square test.

We performed random forest (RF) classification to identify patient and treatment factors predictive of our outcomes. Random forest classification is a machine-learning technique that uses an ensemble of decision trees to predict a categorical outcome. Specifically, we trained three classifiers. We trained the classifiers to predict the achievement of hemodynamic targets, recommended by the 2011 and 2022 guidelines (SBP <120 mmHg or HR <60 bpm), at the time of ED departure. We then trained the classifiers to predict the achievement of hemodynamic targets recommended by the updated 2022 AHA guidelines (HR <80 bpm). We selected 22 predictors a priori for these models including variables related to age, past medical history and outpatient medications, ED triage vital signs and laboratory values, diagnostic evaluation, and treatment (Appendix 1). While our retrospective analysis could only demonstrate the association, the RF algorithm does not provide measurements of association such as odds ratios or risk ratios as traditional regression models do. The machine-learning algorithms such as RF instead reported “predictors,” which referred to clinical factors that would be statistically associated with the outcomes, in this retrospective context.

The results from the RF analyses were depicted as dot plots. The Y-axis represents the order of significant contributions from top (most significant) to bottom (least significant). The X-axis represents the Shapley additive explanations (SHAP) values. The SHAP values use a game-theoretic approach to assign how much a specific feature of a model contributes to its outcome. For our models, positive SHAP values indicated predictors not meeting the AHA guidelines (patients having SBP >120 mmHg or HR >60 bpm, HR >80 bpm at ED departure), while predictors with negative SHAP values indicated patients meeting AHA guidelines (having SBP <120 mmHg, HR <60 bpm). The magnitude of these values corresponds to the extent to which the feature contributes to the model prediction.

We assessed performances of the RF models via the accuracy test, with values approaching 1.0 indicating good discriminatory capability of the models. Sensitivity and specificity were also reported for each RF model. We performed all analyses using Python v3.10.12 (Python Software Foundation, Wilmington, DE). Random forest classifiers and SHAP values were generated using the Python sklearn (v1.4.2) and SHAP (v0.44.1) libraries, respectively. All analyses with a 2-tailed *P*-value < 0.05 were considered statistically significant.

## RESULTS

**Figure 1:**
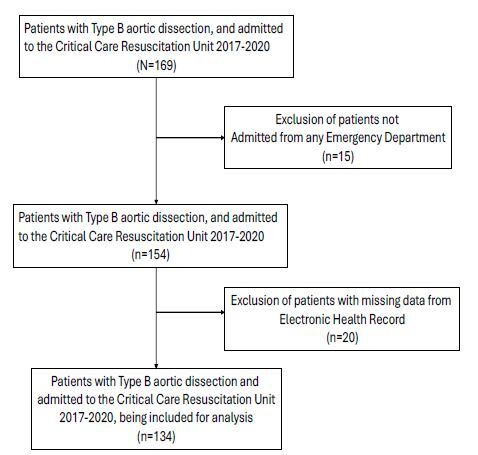
Patient selection flow diagram *CCRU*, critical care resuscitation unit; *ED*, emergency department; *EHR*, electronic health record.

We included 134 patients with type B aortic dissection (Figure 1); all patients were transferred from other hospitals’ EDs to our resuscitation unit. The mean SBP (+/− SD) at ED triage was 157 ± 41 mmHg and the mean SBP at referring ED departure was 145 ± 35 mmHg. The mean HR at ED triage and referring ED departure was 78 ± 18 bpm and 76 ± 16 bpm, respectively (Table 1). All patients’ blood pressure measurements were from non-invasive cuff blood pressure measurements. Twenty-six (19%) patients achieved SBP <120 mmHg prior to referring ED departure, 16 (12%) HR <60 bpm (Table 2A), and 88 (66%) HR <80 bpm (Table 2B). Three (2%) patients achieved both HR and SBP within the parameters recommended by the 2010 AHA guidelines (SBP <120 mmHg and HR < 60bpm), while 15 (11%) achieved both parameters as recommended by the 2022 guidelines (SBP <120 mmHg and HR <80 bpm).

Ninety-three patients (69%) received impulse control therapy while in the ED: 44 (33%) received IV beta blockers, and 10 (8%) received IV vasodilators in the form of hydralazine or nitroglycerin tablets. A total of 89 (62%) were treated with AHT infusions, 49 (37%) received esmolol, 38 (28%) received either nicardipine or clevidipine, and 2 (1%) patients received nitroprusside. Sixteen (12%) received both esmolol and either nicardipine or clevidipine. A total of 82 patients received opioid medications for pain control. The average morphine equivalent units (MEU) of administered opioids were 10.05 ± 7.69. Fifty-two (36%) patients ultimately underwent surgical intervention for type B aortic dissection during their index hospitalization following transfer to UMMC.

### Primary Outcome: Systolic Blood Pressure <120 mmHg

**Figure 2:**
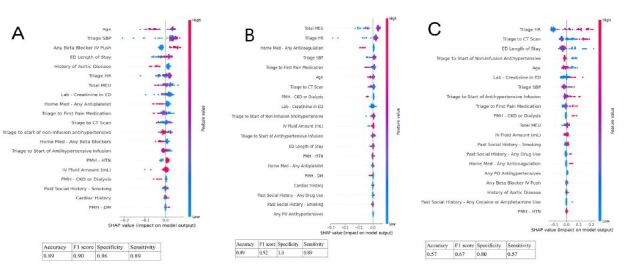
Dot plots generated from random forest analysis for the outcomes of interest (Figure A for SBP < 120 mm Hg; B for HR <60 bpm; C for HR <80 bpm). Blue dots represent lower values of the predictors, while red dots represent higher values of the predictor. Dots to the left of the vertical midline represented negative SHAP values, with prediction for SBP <120 mmHg. *CKD*, chronic kidney disease; *CT*, computed tomography; *DM*, diabetes mellitus; *ED*, emergency department; *HR*, heart rate; *HTN*, hypertension; *IV*, intravenous; *PMH*, past medical history; *MEU*, morphine equivalent unit; *mL*, milliliter; *PO*, per oral; *SBP*, systolic blood pressure.

Twenty-six (19%) patients had SBP <120 mmHg at departure from the referring EDs (Table 1). The RF algorithm identified older age (SHAP −0.012) as the top predictor for SBP <120 mmHg, as well as higher triage SBP (SHAP 0.018) and higher triage HR (SHAP 0.0009) as top predictors for SBP >120 mmHg at ED departure (Table 3) (Figure 2A). Patients with a shorter ED length of stay were also more likely to have SBP >120 mmHg at ED departure (SHAP −0.0042). This model had good performance (accuracy = 0.89, F1 score = 0.90, specificity = 0.96, sensitivity = 0.89).

### Secondary Outcome: Heart Rate <60 bpm at ED Departure (time at leaving the referring EDs)

Sixteen (12%) patients had HR <60 bpm at ED departure (Table 2A). Total MEU, triage HR, triage SBP, and time from triage to first pain medication were among the top predictors for HR <60 bpm at ED departure. Patients receiving pain medications with a higher total MEU (SHAP −0.0015) were more likely to achieve HR <60 bpm at ED departure, while those with higher triage HR (SHAP 0.003) and higher triage SBP (SHAP 0.001) were more likely to have a HR >60 bpm at ED departure (Table 3) (Figure 2B).

### Secondary Outcome: Heart Rate <80 bpm at ED Departure (time at leaving the referring EDs)

Eighty-eight (66%) patients had HR <80 bpm at ED departure (Table 2B). Triage HR, triage SBP, ED length of stay, time from triage to CT scanner, and age were among the top predictors for HR <80 bpm at ED departure. Patients with lower triage SBP (SHAP −0.015) and longer ED length of stay (SHAP −0.0008) were more likely to achieve HR <80 bpm at ED departure, while those with higher triage HR (SHAP 0.002), longer time from triage to CT scanner (SHAP 0.011), and younger age (SHAP 0.005) were more likely to have HR >80 bpm at ED departure (Table 3) (Figure 2C).

## DISCUSSION

This study identified a number of predictors for patients with type B aortic dissection who met AHA guidelines for SBP and HR at the time of leaving the referring ED prior to transfer to a quaternary center. The majority of patients who presented with type B aortic dissections to EDs did not meet 2010 AHA goals for either SBP (19%) or HR (12%) during their ED stays, and only three patients (2%) met both goals. This appears to be similar to the findings in previous literature.^11^,^12^ The majority of patients (66%) achieved the more liberal HR goal recommended by the updated 2022 AHA guidelines, although these updated guidelines were not yet published at the time when they received their care. There was a higher number of patients (11%) who achieved both HR and SBP parameters as recommended by 2022 guidelines.

Patients who did achieve SBP <120 mmHg at ED discharge presented with lower average triage SBP and had a shorter time from triage to CT, potentially allowing clinicians to more rapidly diagnose the aortic pathology and initiate medical management while awaiting transfer to higher level of care. While these findings did not achieve statistical significance, we observed a trend toward more rapid administration of the first dose of AHT infusion and initiation of continuous infusions among patients who achieved SBP < 120 mmHg at leaving the ED prior to transfer to a quaternary care center. Our analysis also suggested that patients who received a higher number of IV beta-blocker pushes were not likely to achieve SBP <120 mmHg at ED departure (Figure 2A). While we could not exclude the reverse association that patients with higher SBP would receive more doses of IV beta blocker, the combination of early AHT infusion and ineffectiveness of IV push beta blocker may present a potential modifier for clinical change for clinicians. Prior studies have demonstrated a benefit of early initiation of nicardipine infusions and improved efficacy of nicardipine when compared to IV labetalol in patients with acute intracranial hemorrhage or stroke.^14^,^15^ Based on our findings, we suggest clinicians have a low threshold to escalate impulse control therapy to include beta-blocker and AHT infusions in patients with acute type B dissections not meeting the hemodynamic parameters recommended by the AHA.

Our findings further support the recommendation by the AHA that pain management be considered a component of hemodynamic management in patients with aortic dissection. Our analysis identified a higher total MEU as a predictor for HR <60 bpm at the time of transferring from the ED to a quaternary care center. Aortic dissections cause significant pain, which not only heightens patient suffering but also contributes to hypertension and tachycardia. It has been suggested that opioids specifically can attenuate sympathetic alpha-adrenergic outflow, thereby functioning as an additional component of anti-impulse therapy.^16^ In our study, just over half of the population received any form of pain medication.

While there is certainly a subset of patients with type B aortic dissections who require surgical management (and this proportion was relatively high in our population [35%] likely due to our selection of complex patients at high risk of need for repair as priorities for transfer to our quaternary care center), the acute management, and for many patients, the primary treatment of the disease is medical. This anti-impulse therapy can and should be started in the ED immediately following diagnosis, as the appropriate titration of medications requires time, and their impact is not immediate. We found that longer ED length of stay and shorter time from ED triage to CT were key drivers of achieving HR goals as recommended by the AHA, suggesting that with increased time after diagnosis, emergency clinicians were able to make significant progress. In addition to securing a disposition, appropriate, aggressive, and timely management of aortic disease within the ED is only becoming more important as ED boarding, including of critically ill patients, becomes more the norm than an anomaly.

## LIMITATIONS

As this was a single-center study, it is difficult to generalize our findings. At UMMC, the vascular surgery team is responsible for management of type B dissection patients who are transferred from any referring hospitals. As a referral center with expertise in open and endovascular repair of complex dissections requiring surgical interventions, a relatively large proportion of our patients are managed surgically, given that the high likelihood of need for surgical intervention is most often considered the indication for transfer. Thus, the successful medical management of these patients may be more challenging than that of patients with a lower rate of ultimate surgical intervention, although conversely, patients with poor hemodynamic control throughout the hospitalization course may have also been at higher risk for requiring surgical management.

During our data collection, we lost a significant number of patients due to lack of access to their ED records from outside facilities directly before transfer, which was consistent with previous observations that documentation for patients with dissections were somewhat inadequate by both emergency clinicians^17^ and in transport,^18^ likely due to patients’ acuity and familiarity with the disease state. We were unable to account for the vitals or management of these patients during transport due to limited documentation from transport clinicians in the EHR. Additionally, when predicting outcomes based on AHA guidelines, the training data for our RF classifiers only had a few patients who met the criteria. Unproportional examples of these outcomes likely led to bias and variance imbalance, which could limit the generalizability of these models.

## CONCLUSION

Many patients transferred from EDs to a quaternary care center for management of type B aortic dissections did not achieve hemodynamic parameters in line with either the 2010 or 2022 American Heart Association guidelines prior to transfer. Patients with higher HR and SBP on ED arrival were less likely to achieve goals at the time of leaving the referring ED, while those who received more pain medications, prior to transfer, were more likely to meet certain AHA goals. Emergency physicians should be cognizant of this patient population given the high morbidity and mortality that type B dissections present with. Antihypertensive therapy and opioid pain medications should be prioritized in these patients as soon as the diagnosis is made and should be continued even through transport to continue to achieve AHA guidelines.

## Supplementary Information





## Figures and Tables

**Table 1:** Comparison of characteristics of patients who presented to the emergency department (ED) with type B aortic dissection and left the ED with SBP < 120 mmHg vs those who left the ED with SBP > 120 mmHg.

Variables	All patients N = 134	SBP <120 mmHg n = 26	SBP >120 mmHg n = 108	Difference between groups	95% CI of differences	P-value
Age (mean, SD)	64(14)	65(16)	64(13)	0.9	−5.2,6.9	0.78
Male (N,%)	84(63)	13(50)	71(66)	−0.2	−0.4,0.0	0.14
Female (N,%)	50(37)	13(50)	37(34)	0.2	0.0,0.4	0.14
PMH - DM (N,%)	13(10)	0(0)	13(12)	−0.1	−0.2,0.0	0.06
PMH - HTN (N,%)	102(76)	17(65)	85(79)	−0.1	−0.3,0.0	0.15
PMH - CKD or dialysis (N,%)	11(8)	2(8)	9(8)	0.0	−0.1,0.2	0.91
Cardiac history (N,%)	22(16)	3(12)	19(18)	−0.1	−0.2,0.1	0.45
History of aortic disease (N,%)	30(22)	11(42)	19(18)	0.2	0.1,0.4	0.01
Clinical information						
Triage SBP, (mmHg) (mean, SD)	158(40.9)	133(45.8)	163(37.5)	−30.4	−47.4, −13.5	0.0
Leaving ED SBP, (mmHg) (mean, SD)	145(34.5)	101(16.5)	156(28.7)	−54.9	−66.5, −43.3	0.0
Triage HR (bpm) (mean, SD)	78(17.6)	81(25.3)	77(15.2)	4.1	−3.5,11.8	0.28
Leaving ED HR (bpm) (mean, SD)	75(15.5)	76(13.1)	75(16.1)	1.0	−5.7,7.8	0.76
Triage HR (bpm) (median, IQR)	76[66.5–91]	81[64.3–95]	74[66.5–89.5]	6.5	−6.5,16.0	0.50
Triage pain (median, IQR)	7.0[4–10]	6.5[0–9.5]	8.0[4–10]	−1.5	−8.0,2.0	0.29
Time of treatment while in the emergency department						
Triage to first antihypertensive (hours) (median, IQR)	3.0[1.5–4.7]	1.9[0.9–3.2]	3.0[1.5–4.9]	−1.2	−2.5,0.6	0.10
Triage to start of antihypertensive infusion (hours) (median, IQR)	3.2[1.8–4.7]	3.0[1.8–3.3]	3.3[1.8–4.9]	−0.3	−1.9,0.7	0.33
Triage to first pain medication (hours) (median, IQR)	1.05[0.5–2.]	1.4[0.7–2.1]	1.0[0.5–2]	0.3	−0.6,1.2	0.60
Triage to CT (hours) (median, IQR)	2.3[1.5–4]	1.6[1.3–2.5]	2.5[1.5–4.1]	−0.9	−1.6,0.1	0.06
Treatment in the emergency department						
Any infusions (N,%)	55(41)	8(31)	47(44)	−0.1	−0.3,0.1	0.24
Any esmolol infusion (N,%)	49(37)	8(31)	41(38)	−0.1	−0.2,0.1	0.49
Any CCB infusion (N,%)	38(28)	2(8)	36(33)	−0.3	−0.4,−0.1	0.01
Both esmolol and CCB infusion (N,%)	16(12)	1(4)	15 (11)	−0.1	−0.2, 0.0	0.046
Nitroglycerin (N,%)	5(4)	1(4)	4(4)	0.0	−0.1,0.2	0.97
Nitroprusside (N,%)	2(1)	0(0)	2(0)	0.0	−0.1,0.1	0.48
Any IV push (N,%)	46(34)	3(12)	43(40)	−0.3	−0.4,−0.1	0.01
Any beta-blocker IV push (N,%)	44(33)	3(12)	41(38)	−0.3	−0.4,−0.1	0.01
Hydralazine IV push (N,%)	5(4)	0(0)	5(5)	−0.1	−0.1,0.1	0.26
Any PO antihypertensives (N,%)	5(4)	1(4)	4(4)	0.0	−0.1,0.2	0.97
Any opioids (N,%)	82(61)	12(46)	70(65)	−0.2	−0.4,0.0	0.08
Total MEU (median, IQR)	8.0[4.5–12.8]	9.0[4.8–11.5]	8.0[4.5–12.8]	1.0	−4.0,5.0	0.85
Other pain medications (N,%)	1(1)	1(4)	0(0)	0.0	0.0,0.2	0.04
ED length of stay (hours) (median, IQR)	5.6[3.5–8.3]	3.9[3.1–7.9]	5.7[3.8–7.8]	−1.8	−2.9,1.2	0.14

*CCB*, calcium channel blocker; *CKD*, chronic kidney disease; *CT*, computed tomography; *DM*, diabetes mellitus; *ED*, emergency department; *HR*, heart rate; *HTN*, hypertension; *IQR*, interquartile range; *IV*, intravenous; *MEU*, morphine equivalent unit; *mmHg*, millimeter of mercury; *PMH*, past medical history; *PO*, per oral (by mouth); S*BP*, systolic blood pressure.

**Table 2A:** Characteristic of patients who presented to ED with type B aortic dissection and left the ED with HR < 60 bpm.

Variables	All patients N = 134	HR < 60 bpm n = 16	HR >= 60 bpm n = 118	Difference between groups	95% CI of differences	P-value
Age (mean, SD)	64(14.1)	69(9.8)	64(14.5)	4.7	−2.7,12.0	0.22
Male (N,%)	84(63)	12(75)	72(61)	0.1	−0.1,0.3	0.28
Female (N,%)	50(37)	4(25)	46(39)	−0.1	−0.3,0.1	0.28
PMH - DM (N,%)	13(10)	3(19)	10(8)	0.1	0.0,0.4	0.19
PMH - HTN (N,%)	102(76)	14(88)	88(75)	0.1	−0.1,0.3	0.26
PMH - CKD or dialysis (N,%)	11(8)	4(25)	7(6)	0.2	0.0,0.4	0.01
Cardiac history (N,%)	22(16)	0(0)	22(19)	−0.2	−0.3,0.0	0.06
History of aortic disease (N,%)	30(22)	3(19)	27(23)	−0.0	−0.2,0.2	0.71
Clinical information						
Triage SBP, (mmHg) (mean, SD)	158(40.9)	140(36.9)	160(41.0)	−19.5	−40.8,−1.9	0.07
Leaving ED SBP, (mmHg) (mean, SD)	145(34.5)	134 (24.4)	147(35.5)	−12.8	−31.0,5.3	0.16
Triage HR, (bpm) (mean, SD)	78(17.6)	58(10.2)	81(16.6)	−22.9	−31.3,14.4	0.00
Leaving ED HR (bpm) (mean, SD)	75(15.5)	52(12.9)	78(13.1)	−26.1	−33.0,−19.3	0.00
Triage SBP (median, IQR)	151[132–180]	130[116.8–171.8]	152[135–185]	−22.0	−39.0,22.0	0.06
Triage HR (bpm) (median, IQR)	76[66.5–91]	56[51.3–64]	80[69–92]	−24.5	−28.5,−13.5	0.00
Triage pain (median, IQR)	7.0[4–10]	8.0[5–9]	7.0[4–10]	1.0	−4.0,2.5	0.89
Treatment while in the emergency department						
Triage to first antihypertensive (hours) (median, IQR)	3.0[1.5–4.7]	2.1[0.7–3.8]	3.0[1.5–4.7]	−0.9	−2.5,2.5	0.41
Triage to start of antihypertensive infusion (hours) (median, IQR)	3.2[1.8–4.7]	3.1[2.1–5.1]	3.0[1.8–4.5]	0.1	−1.8,3.1	0.91
Triage to first pain medication (hours) (median, IQR)	1.1[0.5–2]	1.7[0.9–2]	0.9[0.5–2]	0.8	−0.2,1.2	0.12
Triage to CT (hours) (median, IQR)	2.3[1.5–4]	2.4[1.6–3.4]	2.3[1.5–4]	0.1	−0.9,1.3	0.96
Treatment in the emergency department						
Any infusions (N,%)	55(41)	5(31)	50(42)	−0.1	−0.3,0.2	0.40
Any esmolol infusion (N,%)	49(37)	3(19)	46(39)	−0.2	−0.4,0.1	0.11
Any CCB infusion (N,%)	38(28)	4(25)	34(29)	0.0	−0.2,−0.2	0.75
Nitroglycerin (N,%)	5(4)	3(19)	2(2)	0.2	0.0,0.4	0.00
Nitroprusside (N,%)	2(1)	0(0)	2(2)	0.0	−0.1,0.2	0.60
Any IV push (N,%)	46(34)	3(19)	43(36)	−0.2	−0.3,0.1	0.16
Any beta-blocker IV push (N,%)	44(33)	3(19)	41(35)	−0.2	−0.3,−0.1	0.20
Hydralazine IV push (N,%)	5(4)	1(6)	4(3)	0.0	0.0,0.3	0.57
Any PO antihypertensives (N,%)	5(4)	0(0)	5(4)	0.0	−0.1,0.2	0.40
Any opioids (N,%)	82(61)	13(0.81)	69(58)	0.2	0.0,0.4	0.08
Total MEU (median, IQR)	8.0[4.5–12.8]	5.0[4–8]	8.0[5–13.5]	−3.0	−6.0,0.0	0.04
Other pain medications (N,%)	1(1)	0(0)	1(1)	0.0	−0.1,0.2	0.71
Any vascular surgeries during hospital stay (N,%)	71(53)	12(75)	59(50)	0.3	0.0,0.4	0.06
Hospital length of stay (days), (median, IQR)	7.4[4–12]	6.4[4.9–8.9]	7.7[3.9–12]	−1.3	−3.5,1.2	0.48
Mortality (N,%)	11(8)	2(12)	9(8)	0.0	−0.1,0.3	0.51
ED length of stay (hours) (median, IQR)	5.6[3.5–8.3]	4.8[3.5–9.2]	5.6[3.5–7.8]	−0.8	−2.3,3.4	0.94

*CCB*, calcium channel blocker; *CKD*, chronic kidney disease; *CT*, computer tomography; *DM*, diabetes mellitus; *ED*, Emergency Department; *HR*, heart rate; *HTN*, hypertension; *IQR*, interquartile range; *IV*, intravenous; *MEU*, morphine equivalent unit; mmHg, millimeter of mercury; *PMHx*, past medical history; *PO*, per oral (by mouth); *SBP*, systolic blood pressure.

*HR*, heart rate; *bpm*, beats per minute; *CI*, confidence interval; *IRQ*, interquartile range; *ED*, emergency department.

**Table 2B:** Characteristics of patients who presented to emergency department (ED) with type B aortic dissection and left the ED with heart rate <80 bpm.

Variables	All patients N = 134	HR < 80 bpm n=88	HR ≥ 80 bpm n = 46	Difference between groups	95% CI of differences	P-value
Age (mean, SD)	64(14.1)	65(13.5)	63(15.1)	2.1	−3.0,7.2	0.41
Male (N,%)	84(63)	58(66)	26(57)	0.1	−0.1,0.3	0.29
Female (N,%)	50(37)	30(34)	20(43)	−0.1	−0.3,0.1	0.29
PMH - DM (N,%)	13(10)	8(9)	5(11)	0.0	−0.2,0.1	0.74
PMH - HTN (N,%)	102(76)	65(74)	37(80)	−0.1	−0.2,0.1	0.40
PMH - CKD or dialysis (N,%)	11(8)	5(6)	6(13)	−0.1	−0.2,0.0	0.14
Cardiac history (N,%)	22(16)	14(16)	8(17)	0.0	−0.2,0.1	0.83
History of aortic disease (N,%)	30(22)	18(20)	12(26)	−0.1	−0.2,0.1	0.46
Clinical information						
Triage SBP, (mmHg) (mean, SD)	158(40.9)	156(37.3)	160(47.4)	−3.3	−18.1,11.4	0.66
Leaving ED SBP, (mmHg) (mean, SD)	145(34.5)	142(30.0)	152(41.5)	−9.5	−21.9,2.8	0.13
Triage HR, (bpm) (mean, SD)	78(17.6)	72(16.0)	90(14.2)	−18.2	−23.7,−12.6	0.00
Leaving ED HR (bpm) (mean, SD)	75(15.5)	67(10.1)	91(10.8)	−24.4	−28.1,20.6	0.00
Triage SBP (median, IQR)	151[132–180]	152[133.5–180]	148[129.8–189.8]	4.5	−21.0,17.0	0.97
Triage HR (bpm) (median, IQR)	76[66.5–91]	70.0[62.5–79.5]	90[80.3–100]	−19.5	−26.5,−13.6	0.00
Triage pain (median, IQR)	7.0[4–10]	8.0[4–10]	6.5[3–8.8]	1.5	−0.5,4.0	0.11
Treatment while in the emergency department						
Triage to first antihypertensive (hours) (median, IQR)	3.0[1.5–4.7]	3.1[1.3–4.8]	2.7[1.8–4.1]	0.4	−0.7,1.5	0.72
Triage to start of antihypertensive infusion (hours) (median, IQR)	3.2[1.8–4.7]	3.0[1.6–4.6]	3.5[1.9–4.5]	−0.5	−1.3,1.2	0.60
Triage to first pain medication (hours) (median, IQR)	1.1[0.5–2]	0.9[0.5–1.9]	1.5[0.7–2.4]	−0.6	−1.4,0.3	0.27
Triage to CT (hours) (median, IQR)	2.3[1.5–4]	2.4[1.6–3.9]	2.1[1.3–4]	0.3	−0.9,1.3	0.59
Treatment in the emergency department						
Any infusions (N,%)	55(41)	36(41)	19(41)	0.0	−0.2,0.2	0.96
Any esmolol infusion (N,%)	49(37)	32(36)	17(37)	0.0	−0.2,0.2	0.95
Any CCB infusion (N,%)	38(28)	24(27)	14(30)	0.0	−0.2,0.1	0.70
Nitroglycerin (N,%)	5(4)	5(6)	0(0)	0.1	0.0,0.1	0.10
Nitroprusside (N,%)	2(1)	2(2)	0(0)	0.0	−0.1,0.1	0.30
Any IV push (N,%)	46(34)	28(32)	18(39)	−0.1	−0.2,0.1	0.40
Any beta-blocker IV push (N,%)	44(33)	28(32)	16(35)	0.0	−0.2,0.1	0.73
Hydralazine IV push (N,%)	5(4)	3(3)	2(4)	0.0	−0.1,0.1	0.79
Any PO antihypertensives (N,%)	5(4)	4(5)	1(2)	0.0	−0.1,0.1	0.49
Any opioids (N,%)	82(61)	58(66)	24(52)	0.1	0.0,0.3	0.12
Total MEU (median, IQR)	8.0[4.5–12.8]	8.0[4.3–13.9]	8.0[5–10]	0.0	−2.5,3.5	0.88
Other pain medications (N,%)	1(1)	1(1)	0(0)	0.0	−0.1,0.1	0.47
Any vascular surgeries during hospital stay (N,%)	71(53)	48(55)	23(50)	0.1	−0.1,0.2	0.62
Hospital length of stay (days), (median, IQR)	7.4[4–12]	7.1[3.9–12]	7.9[4.4–11]	−0.8	−2.9,1.6	0.60
Mortality (N,%)	11(8)	7(8)	4(9)	0.0	−0.1,0.1	0.88
ED length of stay (hours) (median, IQR)	5.6[3.5–8.3]	5.7[3.7–8.3]	4.6[3.3–7.4]	1.1	−1.3,2.3	0.32

*CKD*, chronic kidney disease; *DM*, diabetes mellitus; *ED*, emergency department; *HR*, heart rate; *HTN*, hypertension; *IQR*, interquartile range; *mmHg*, millimeter of mercury; *PMH*, past medical history; *SBP*, systolic blood pressure.

*CT*, computed tomography; *ED*, emergency department; *HR*, heart rate; *IQR*, interquartile range; *IV*, intravenous; *MEU*, morphine equivalent unit; *mmHg*, millimeter of mercury; *PO*, per oral (by mouth); *SBP*, systolic blood pressure.

**Table 3:** Top five features of the random forest analysis for the three outcomes of interest. Each feature contributes to the predictability of the model. The significance of each feature is calculated by the mean absolute SHAP values across all data points.

	SBP <120	HR <60	HR<80
Accuracy	0.89	0.89	0.57
F1 score	0.90	0.92	0.67
Specificity	0.96	1.00	0.8
Sensitivity	0.89	0.89	0.57
Feature 1	Age (−0.012)	Total MEU (−0.001)	Triage HR (0.002)
Feature 2	Triage SBP (0.018)	Triage HR (0.0002)	Time from triage to CT (0.011)
Feature 3	Any beta-blocker IVP (0.002)	Home medication – Any anticoagulation (−0.004)	ED length of stay (−0.0008)
Feature 4	ED length of stay (−0.004)	Triage SBP (0.001)	Time from triage to start of non-infusion anti-hypertensive medication (0.004)
Feature 5	History of aortic disease (−0.0005)	Time from triage to first pain medication (0.001)	Age (0.005)

*CT*, computed tomography; *ED*, emergency department; *HR*, heart rate; *IVP*, intravenous push; *MEU*, morphine equivalent unit; *SBP*, systolic blood pressure; *SHAP*, Shapley additive explanations.
