# Supplementary material for: Emergency Department Blood Pressure Management in Type B Aortic Dissection: An Analysis with Machine Learning
Source: West J Emerg Med. 2025 May 5;26(3):674–84. doi: 10.5811/westjem.25005 (PMC12208080; doi:10.5811/westjem.25005)
Supplement: Supplementary file 1 [file wjem-26-674-s001.docx]

**Appendix 1**. List of variables being used for all Random Forest analyses. All variables were selected a priori and were entered into the models.

| **Predictor** |
| --- |
| Age |
| History of Hypertension (HTN) |
| Diabetes (DM) |
| Chronic Kidney Disease (CKD) or dialysis |
| Known Cardiovascular Disease (CVD) or aortic disease |
| Home beta blockers, antiplatelets (AP) and/or anticoagulants (AC) |
| History of smoking, any illicit drug use, specifically cocaine or amphetamine use |
| Vital signals (SBP, HR) at ED triage |
| Initial serum creatinine levels on ED labs |
| Administration of IV beta blocker, beta blocker or vasodilator infusion, PO antihypertensives (including ***) in the ED |
| Total amount (in morphine milligram equivalents (MEU)) of all opioids, and IVF (in milliliters (mL)) administered in the ED |
| Time from triage to CT scan |
| Time from triage to start of antihypertensive infusion,  non-infusion antihypertensive, and pain medication |
| Total ED stay time |
