# Supplementary material for: Emergency Department Blood Pressure Management in Type B Aortic Dissection: An Analysis with Machine Learning
Source: West J Emerg Med. 2025 May 5;26(3):674–84. doi: 10.5811/westjem.25005 (PMC12208080; doi:10.5811/westjem.25005)
Supplement: Supplementary file 2 [file wjem-26-674-s002.docx]

**Appendix 2**. The SHapley Additive exPlanations (SHAP) values for predictors in the Random Forest analysis for the 3 outcomes of interest (SBP < 120 mmHg, HR < 60 bpm, HR < 80 bpm). The analyses included all variables in the models.

|  | Outcomes | | |
| --- | --- | --- | --- |
| Variables | SBP <120 | HR <60 | HR <80 |
| Age | -0.012 | -0.005 | 0.005 |
| PMH - HTN | 0.003 | -0.001 | 0.0007 |
| PMH - DM | -0.0002 | -0.00001 | -0.0002 |
| PMH - CKD or dialysis | -0.0008 | -0.002 | 0.003 |
| Any cardiac history | -0.0004 | -0.001 | 0.00003 |
| History of aortic disease | -0.0005 | 0.0006 | 0.0003 |
| Home med - any beta blockers | -0.002 | -0.0006 | 0.001 |
| Home med - any antiplatelet | 0.0008 | -0.002 | -0.0003 |
| Home med - any anticoagulation | -0.0007 | -0.004 | 0.0006 |
| Past social history - smoking | -0.001 | -0.001 | -0.002 |
| Past social history - any drug use | -0.0006 | -0.001 | -0.003 |
| Past social history - any cocaine or amphetamine use | -0.0003 | -0.0001 | -0.0002 |
| Any Beta Blocker IV push | 0.002 | -0.0005 | 0.0007 |
| Any PO antihypertensives | 0.001 | 0.001 | 0.001 |
| Triage SBP | 0.019 | 0.001 | -0.015 |
| Triage HR | 0.0009 | 0.003 | -0.012 |
| Serum creatinine levels in ED | 0.008 | -0.003 | -0.012 |
| Total MEU | 0.004 | -0.002 | -0.0008 |
| IV fluid amount (mL) | -0.003 | -0.000015 | 0.0006 |
| Triage to start of antihypertensive infusion | -0.002 | 0.0004 | -0.003 |
| Triage to first pain medication | -0.006 | 0.001 | 0.002 |
| Triage to CT scan | -0.005 | -0.002 | 0.011 |
| ED length of stay | -0.004 | -0.0009 | -0.009 |
| Triage to start of any antihypertensive infusion | -0.002 | 0.001 | 0.004 |

CT, computer tomography; ED, Emergency Department; HR, heart rate; IV, intravenous; mL, millimeter; MEU, morphine equivalent unit; PO, per oral (by mouth); SBP, systolic blood pressure;
